# How do idiosyncratic deals influence innovation performance? From the perspective of coworker

**DOI:** 10.3389/fpsyg.2022.1091613

**Published:** 2022-12-23

**Authors:** Chen Ding, Lingxiao Deng, Jing Yang, Jiayun Chai

**Affiliations:** ^1^School of Business, Nanjing University, Nanjing, China; ^2^AIEN Institute, Shanghai Ocean University, Shanghai, China; ^3^School of Public Administration and Policy, South China University of Technology, Guangzhou, China; ^4^School of Business, Jiangxi Normal University, Nanchang, China

**Keywords:** idiosyncratic deals, thriving at work, learning, vitality, innovation performance, employee humility

## Abstract

In the hypercompetitive marketplace, contemporary organizations incorporate the diversity of talents into job design (i.e., offering idiosyncratic deals), in order to meet the unique needs of talented employees and achieve the purpose of attracting, motivating, and retaining them. Based on the cognitive-affective processing system framework, this study aims to explore the effect of coworkers’ perceptions of employees’ idiosyncratic deals (CPEID) on coworker innovation performance, the mediating role of thriving at work, and the moderating role of humility. Two-wave data were obtained from 248 employees of 15 China firms. The findings suggest that (a) CPEID increase coworker innovation performance by fostering coworker learning; (b) CPEID decrease coworker innovation performance by undermining coworker vitality; (c) Coworker humility not only positively moderates the relationship between CPEID and coworker learning, but also positively moderates the indirect effect of coworker learning between CPEID and coworker innovation performance; and (d) the moderating role of coworker humility is not significant in the relationship between CPEID and coworker vitality. This study provides a theoretical explanation for whether CPEID have both positive and negative effects on coworker innovation performance, and extends boundary conditions of idiosyncratic deals (i-deals). Besides, the findings inspire managers to make reasonable use of the positive role of i-deals.

## 1. Introduction

In the hypercompetitive marketplace, talented employees have changed their weak position in employment relationships, due to their high bargaining power (Rousseau, 2001). To rebalance employment relationships, contemporary organizations incorporate the diversity of talents into job design, namely offering idiosyncratic deals (i-deals), e.g., flexible work schedules, special training opportunities, and greater advancement opportunities. In doing so, organizations not only meet the unique needs of talented employees but also achieve the purpose of attracting, motivating, and retaining them (Vidyarthi et al., 2016).

I-deals refer to “voluntary, personalized agreements of a nonstandard nature negotiated between individual employees and their employers regarding terms that benefit each party” (Rousseau et al., 2006). Specifically, their negotiations include promotion opportunities, flextime, flex place, job security, and material incentives (Huo et al., 2014; Ng and Lucianetti, 2016). Coworkers’ perceptions of employees’ i-deals (CPEID) refer to coworkers’ active perceptions of whether and to what extent employees obtain i-deals (Hornung et al., 2008; Ng and Feldman, 2010). By carefully observing or gathering information to know the movements of i-dealers, coworkers will obtain various active perceptions used to evaluate their own organizational status, which in turn influences their attitudes and behaviors (Vidyarthi et al., 2016). Coworkers, as the majority of the organization members, whose attitudes and behaviors will affect the ultimate implementation effectiveness of i-deals (Rousseau et al., 2006). Therefore, the key to whether organizations can treat employees fairly and differently lies in whether the implementation of i-ideals can achieve the effectiveness that all three parties (i-dealers, managers, and coworkers) view them as win-win-win or at least win-win-no lose (Lai et al., 2009).

Compared to ample research from the receiver’s perspective (i-dealers), the research from the bystander’s perspective (i-dealers’ coworkers) is less. On the one hand, CPEID increase their own expectations of obtaining similar i-deals in the future, which in turn motivates coworkers to engage in organizational citizenship behavior (Huo et al., 2014). On the other hand, CPEID cause coworkers to have negative emotions, which leads to malicious competition and ostracism among employees (Ng, 2017). Prior research had focused on behavioral outcome variables about employees, e.g., voice (Marescaux et al., 2019), helping (Guerrero and Challiol-Jeanblanc, 2016), and work withdrawal (Xiong et al., 2018). However, typical outcome variables about employees were neglected, e.g., innovation performance, and task performance. Given that in-service employees need to make special contributions to the organization in order to get i-deals (Liao et al., 2016), innovation performance not only is a critical work result for employees but also helps employees to make special contributions to the organization, which increases the possibility of obtaining i-deals in the future; in addition, innovation performance is also the best proof of whether the implementation of i-deals can achieve win-win-win or at least win-win-no lose. Based on this, we view coworker innovation performance as the dependent variable to explore the effect of CPEID on coworker innovation performance.

Based on social comparison theory, conservation of resources theory, and equity theory, although prior research explored negative cognition mechanisms, i.e., psychological contract violation (Xiong et al., 2018) and distributive injustice (Marescaux et al., 2019), the promoting effect of CPEID on positive cognition was neglected; though a handful of research explored the mediating role of negative emotions, i.e., envy (Ng, 2017; Wang et al., 2021a) and emotional exhaustion (Kong et al., 2020), they lacked an in-depth discussion on whether CPEID can have both positive and negative effects. Since the events (i.e., i-dealers have received many organizational resources such as i-deals, attention from leaders or organizations, special training opportunities, and job security; Ng and Feldman, 2010) are important to coworkers, coworkers will interpret and evaluate these events. Doing so will directly motivate coworkers’ cognition and affection (Mischel and Shoda, 1995), and ultimately affect their distal outcomes (i.e., attitude, behavior, and performance). Considering this, we subdivide thriving at work into two dimensions of learning and vitality, exploring the double-edged effect of CPEID on coworker innovation performance, based on the cognitive-affective processing system framework. On the one hand, granting i-deals shows that organizations are willing to invest in employees, which will encourage coworkers to regard i-dealers as role models in order to improve their innovation performance through observational learning and advice-seeking. On the other hand, i-dealers occupy important resources of the organization, which reflects that coworkers’ organizational status is impaired; furthermore, this will make coworkers feel nervous, ultimately damaging their innovation performance. Therefore, this study represents learning as the cognitive unit, and vitality as the affective unit.

Owing to CPEID with a double-edge effect, how to strengthen its positive effect and weaken its negative effect are also the focus of this study. Based on individual differences (e.g., individual characteristics, life experiences), when different individuals are confronted with the same event, the idiosyncratic responses of cognition, affection, and behavior will be output, namely individual differences regulate the individual’s encoding process (Mischel and Shoda, 1995). Therefore, coworkers’ individual characteristics explain this process (event—cognition/affection). Considering the cultural differences between China and western countries regarding the understanding of CPEID (Huo et al., 2014), Chinese employees influenced by Chinese traditional culture may have the following humble characteristics: clear self-awareness, appreciation of others’ strengths, and willingness to seek advice with an open mind (Exline et al., 2004; Owens and Hekman, 2012). Therefore, we seek to explore the boundary effect of coworker humility, by representing coworker humility as an individual characteristic. Specifically, coworkers with a high level of humility are comfortable expressing appreciation for i-dealers, and can interpret i-deals as an organizational investment in employees based on competence, which is likely to enhance the positive effect of CPEID; coworkers with a low level of humility, due to unclear self-cognition, believe that i-deals come from managers’ partiality (Wang et al., 2021b), which are likely to increase the negative effect of CPEID.

In conclusion, based on the cognitive-affective processing system framework, we explore the effect of CPEID on coworker innovation performance *via* cognition (learning) or affection (vitality), and examine the moderating role of coworker humility. This study makes managers aware of the double-edged effect of CPEID on coworker innovation performance, and inspires managers reasonably to use the positive role of i-deals.

## 2. Theory and hypothesis

The cognitive-affective processing system framework shows that individuals will activate different cognitive and affective units in the process of evaluating an event (Mischel and Shoda, 1995). Therefore, when coworkers carefully observe and gather information to know the movements of idealers and form a perception used to evaluate their organizational status (Vidyarthi et al., 2016), coworkers will activate different cognitions and affections. Thriving at work is characterized by the joint experience of learning (cognition) and vitality (affection; Spreitzer et al., 2005). A handful of research suggests that the two components (learning and vitality) of thriving at work have differential effects (Prem et al., 2017), and combining the two-wave structures of learning and vitality into a single test of thriving at work can cause bias in the estimation (Guo and Hu, 2022). Therefore, this study subdivides thriving at work into two dimensions of learning and vitality, exploring the effect of CPEID on coworker innovation performance *via* cognition (learning) or affection (vitality), i.e., the positive effect of inspiring coworkers to learn and the negative effect of undermining coworkers’ vitality.

### 2.1. The mediating role of learning

The cognitive-affective processing system framework shows that events affecting an individual’s resources will activate a corresponding cognitive response (Mischel and Shoda, 1995). Therefore, coworkers’ perception that others get i-deals will activate a positive cognitive unit (learning). Learning refers to the cognitive experience that an individual is acquiring, and can apply knowledge and skills (Spreitzer et al., 2005). Specifically, i-dealers obtain many organizational resources (e.g., attention from leaders, special training opportunities, and job security), and can fully utilize their knowledge and skills in the workplace, which shows that organizations are willing to invest in employees (Ng and Lucianetti, 2016). By interpreting this event, coworkers truly feel the approbation of organizations on i-dealers’ competence (Ho and Kong, 2015), and also increase coworkers’ confidence in obtaining similar i-deals in the future, such that they will be willing to improve their competence by learning (Huo et al., 2014). Coworkers regard i-dealers as role models to learn through observation and interaction. In doing so, coworkers gradually recognize i-dealers’ strengths and their own weaknesses, and get effective information on how to improve themselves (Ma et al., 2022), e.g., i-dealers’ workflows, and risky negative behaviors (Lee and Duffy, 2019). Besides observation and imitation, coworkers can also directly interact with i-dealers, e.g., by seeking advice, and asking for feedback (Pan et al., 2021). Considering that i-dealers give coworkers more careful, accurate, and targeted feedback through interaction (Lee and Duffy, 2019), coworkers can receive more direct information input (De Stobbeleir et al., 2011) to gradually close the gap with i-dealers (Wang et al., 2021a).

Learning can enhance coworker innovation performance. On the one hand, the learning process effectively activates the individual’s self-perfection motivation which encourages individuals to actively pursue more achievements and approbations (Pierce and Gardner, 2004), e.g., coworkers will proactively solve organizational problems to gain support from leaders. On the other hand, coworkers willing to learn can positively view their surroundings, and are more willing to engage in interpersonal interactions. Specifically, the coworkers not only will proactively learn or seek help from i-dealers to improve their own knowledge and skill deficiencies, but also expand their own attention span and increase their own activity of thinking in order to enable them to adopt flexible, appropriate work strategies; in addition, interpersonal interaction enhances the relationship between coworkers and i-dealers, which facilitates the rapid dissemination of resources (e.g., knowledge) within the organization, and creates a favorable climate for knowledge sharing (Lee and Duffy, 2019). Therefore, coworkers can improve their own innovation performance by obtaining overflow resources of i-dealers in the cooperative network (Grigoriou and Rothaermel, 2014).

In summary, granting i-deals shows that organizations are willing to invest in their own employees, and set role models for coworkers, which motivates coworkers to learn, and ultimately improves coworker innovation performance. Accordingly, the following hypothesis is proposed.

*Hypothesis* 1: Coworker learning mediates the positive relationship between CPEID and coworker innovation performance.

### 2.2. The mediating role of vitality

According to the cognitive-affective processing system framework (Mischel and Shoda, 1995), besides the positive cognitive unit represented by learning, coworkers’ affective unit represented by vitality will also be activated by the event that others obtain i-deals. Vitality refers to the positive experience of having energy available, reflecting feelings of aliveness (Spreitzer et al., 2005). In order to evaluate their own organizational status, coworkers will proactively collect and covertly observe the movement of i-dealers (Vidyarthi et al., 2016). I-dealers obtained many organizational resources, e.g., attention from leaders, special training opportunities, and job security (Rousseau et al., 2016). Considering that organizational resources are scarce (Wang et al., 2021a) and coworkers are highly sensitive to their own interests (e.g., salary, promotion), i-dealers occupying a large number of organizational resources increase work stress and perceptions of resource threat on coworkers (Ma et al., 2022), which shows that coworkers’ organizational status has been compromised (Liao et al., 2016). Coworkers find themselves in a disadvantageous position by interpreting i-dealers’ movement, which adds to the psychological pressure on coworkers about how to get i-deals. On the one hand, this can lead to negative emotions toward coworkers (e.g., relative deprivation, anxiety, and dissatisfaction). On the other hand, this reduces coworkers’ aspirations for the future and induces them to perceive the uncertainty of obtaining i-deals in the future, which will activate basic anxiety-related neurological processes, arousing negative emotions such as anxiety and depression (Jonas et al., 2014), and in severe cases even triggering depressive reactions (Cohen-Charash, 2009).

Low vitality can decrease coworker innovation performance. On the one hand, coworkers with low vitality will carefully assess their surroundings in order to reduce decision risk and uncertainty, which leads coworkers to do a series of behaviors resulting in lower innovation performance (e.g., adhere to work habits, strive to maintain the status quo, and to avoid or abandon innovative behaviors that may expose their flaws and bring negative consequences). On the other hand, the negative emotion of tension and anxiety reduce coworkers’ ability to control their environment and their confidence in solving work problems (Bakker et al., 2008); specifically, coworkers with insufficient control are more likely to make mistakes at work, and coworkers with emotional exhaustion will fall into a vicious cycle (i.e., neither being willing to seek help nor taking the initiative to change the status quo), which further undermines their thinking and creativity. Campbell et al. (2017) findings suggest that antisocial behaviors are common retaliatory responses to threats. Specifically, faced with a sense of work stress and resource threat caused by i-dealers, coworkers with low vitality tend to vent their emotions through aggressive behaviors (e.g., bullying, intimidating, and slamming). Considering the negative effect of adopting this behavior in the workplace on coworkers’ reputation and status, coworkers have to spend extra time and vigor to adjust their emotions, which will lead to negative effects on their innovation performance.

In conclusion, i-dealers occupying significant organizational resources send a signal that coworkers’ organizational status is compromised. This undermines coworker vitality by making coworkers into negative emotions of anxiety and dissatisfaction, which ultimately decreases their innovation performance. Accordingly, the following hypothesis is proposed.

*Hypothesis* 2: Coworker vitality mediates the negative relationship between CPEID and coworker innovation performance.

### 2.3. CPEID and innovation performance

The cognitive-affective processing system framework shows that the events can activate both cognitive and affective units (Mischel and Shoda, 1995). Therefore, according to H1, H2, and this framework, CPEID can simultaneously activate coworkers learning (cognition) and coworker vitality (affection). A recent study shows that there are many differences between learning and vitality in terms of their effect on innovation performance, i.e., learning is a stronger contributor to innovation than vitality (Guo and Hu, 2022). Specifically, compared to individuals’ emotional responses which are short-lasting and unstable, individuals’ cognitive responses are longer-lasting and more rational (Mischel and Shoda, 1995). Therefore, although CPEID undermine coworker vitality in the short term, it stimulates coworker learning in the long term (Prem et al., 2017). Additionally, given that innovation requires knowledge input, the growth in knowledge from learning is more important than vitality from positive emotions(Guo and Hu, 2022). Taken together, this study speculates that CPEID have a positive total indirect effect on coworker innovation performance. Accordingly, the following hypothesis is proposed.

*Hypothesis* 3: CPEID have a positive total indirect effect on coworker innovation performance via simultaneously motivating coworker learning and vitality.

### 2.4. The moderating role of employee humility

Humility consists of three main dimensions, i.e., accurate self-awareness, appreciation of others’ strengths, and teachability (Owens et al., 2013). Specifically, humble employees (a) have accurate and clear self-awareness and can openly admit their own shortcomings; (b) appreciate the strengths of their coworkers and their contributions to the organization; and (c) are willing to humbly learn new knowledge from coworkers and leaders (Exline et al., 2004; Owens and Hekman, 2012). Based on the cognitive-affective processing system framework, a study shows that there are individual differences in the interpretation and assessment of events, i.e., personality traits affect the process (the activation of cognitive and affective units by events), which in turn influences individuals’ behavioral choices (Mischel and Shoda, 1995). Therefore, this study hypothesized that coworker humility would explain the “event-cognition/affection” process.

When the level of humility is high, coworkers (a) can openly express appreciation for i-dealers; (b) are clearly aware of areas where they are inferior to i-dealers (e.g., knowledge, competence, and experience); (c) are aware of that the organization invests in employees base on employee abilities (Ng and Lucianetti, 2016); and (d) tend to view i-deals as a reasonable measure to improve management efficiency. Based on the above behaviors, coworkers believe a fact that by improving their abilities, they will be able to get i-deals in the future. On the one hand, this increases coworkers’ internal motivation (Prem et al., 2017); specifically, this inspires coworkers to focus on their self-growth and development, and motivates coworkers to take the initiatives to improve their capabilities optimistically. On the other hand, this also encourages coworkers to humbly learn from i-dealers in order to achieve the ability level of obtaining i-deals in the future, e.g., using indirect or direct learning methods (e.g., observing, imitating, and seeking advice; Lee and Duffy, 2019; Pan et al., 2021). Taken together, high levels of humility reinforce the positive effect of CPEID on coworker learning.

When the level of humility is low, coworkers’ self-perceptions are unclear and self-serving, which leads coworkers to selfishly interpret their own and i-dealers’ achievements in order to boast about themselves and devalue i-dealers (Ma et al., 2022). Specifically, when i-dealers have not yet brought clear benefits to the organization, coworkers argue that the allocation of i-deals is most likely the result of managerial bias (Wang et al., 2021a), i.e., i-dealers have interpersonal advantages with leaders rather than objective advantages (e.g., knowledge, skills). Coworkers’ negative interpretation ultimately influences coworker learning and vitality. Specifically, this not only undermines coworker vitality by inducing negative emotions (e.g., anger, self-worth denial, and anxiety; Schmitt et al., 2010), but also inhibits coworker learning by exacerbating coworkers’ negative perceptions of potential harm or loss and reducing coworkers’ motivation to observe, seek advice and imitate. Accordingly, the following hypothesis is proposed.

*Hypothesis* 4: Coworker humility positively moderates the relationship between CPEID and coworker learning/vitality: compared to the low level of coworker humility, the high level of coworker humility can strengthen the positive relationship between CPEID and coworker learning (4a), and weaken the negative relationship between CPEID and coworker vitality (4b).

Based on the above hypotheses, this study suggests that coworker humility, respectively, moderates the indirect effect of learning and vitality between CPEID and innovation performance. When the level of humility is high, coworkers can recognize the initiatives of i-deals and believe that the gap between themselves and i-dealers can be closed through learning, which not only reduces coworkers’ anxiety about the future, but also allows coworkers to be positive and optimistic in their risk assessment (Marescaux et al., 2021). In addition, owing to the expectation of receiving similar treatment in the future, coworkers are willing to improve their efficiency by taking initiatives to ameliorate the existing technology, which ultimately increases innovation performance. However, the low level of humility not only weakens the positive effect of CPEID on coworker learning, but also induces coworker’s low vitality feelings (e.g., hostility, anxiety, resentment, and anger). Therefore, coworkers are likely to choose negative behavioral responses to reduce decision risk and uncertainty (e.g., by avoiding or abandoning innovation), which ultimately undermines coworker innovation performance. Accordingly, the following hypothesis is proposed.

*Hypothesis* 5: Coworker humility moderates the mediating effect of coworker learning/vitality between CPEID and coworker innovation performance: compared to the low level of coworker humility, the high level of coworker humility can strengthen the mediating effect of coworker learning between CPEID and coworker innovation performance (5a), and weaken the mediating effect of coworker vitality between CPEID and coworker innovation performance (5b).

**Figure 1:**
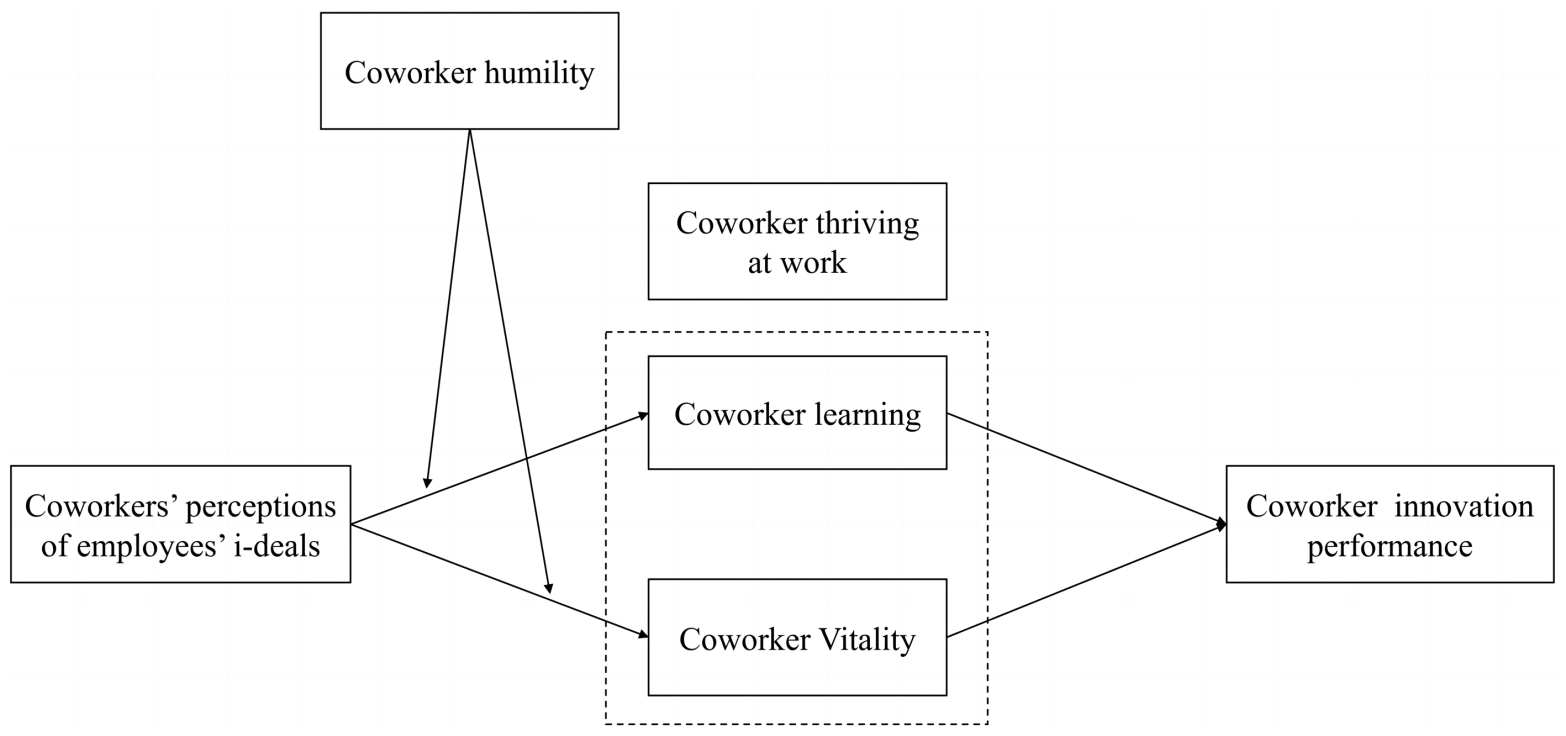
Theoretical model.

In summary, the conceptual model used in this study is shown in Figure 1.

## 3. Materials and methods

### 3.1. Participants and procedure

Considering that knowledge employees have better chances to get i-deals (Wang et al., 2021a), our data were collected from questionnaire surveys on knowledge employees in product development departments of 15 high-tech enterprises from four cities (i.e., Wuhan, Hangzhou, Nanjing, and Guangzhou). Given that whether employees’ own have i-deals is an important boundary condition for CPEID to influence coworkers’ behavior (Ng, 2017), this study investigated the employees who did not obtain i-deals. With the assistance of alumni, we verified with corporate managers that the company had i-deals management policies, and obtained the assistance of HRM departments. In order to ensure the accuracy of questionnaire matching across time tags and that each group of employees was from the same team which only have one i-dealer, HRM departments assigned numbers to the employees who volunteered to participate in the survey. To ease employees’ concerns for the survey, we thoroughly explained the goal and guide of the questionnaire, and highlighted that the survey data used for scientific research is completely confidential.

This study adopted a two-wave survey distributed and collected on-site. At Time 1, the total of 300 employees provided information (i.e., perceptions to i-dealer, humility, and demographic characteristics); after eliminating missing data, our final sample consisted of 277 employees (response rate = 92.33%). At Time 2, a second-wave on-site survey was conducted, namely required employees to report information (i.e., thriving at work, innovation performance, and demographic characteristics). after eliminating missing data, our final sample consisted of 248 employees (response rate = 89.53%). Among them, 44.35% of participants were female; 55.65% of participants were male; 86.69% of participants were between 26 and 35 years old; 83.06% of participants’ tenure were under 5 years; 88.71% of participants had a bachelor’s degree or higher; and 72.98% of participants had an income between CNY 5,000 and CNY 9,000.

### 3.2. Measures

Survey items back-translated following Brislin (1970) procedure were completed on a seven-point Likert scale ranging from 1 (not at all) to 7 (to a great extent), and validated repeatedly in the Chinese context.

*Coworkers’ perceptions of employees’ i-deals* (*Time 1*). Drawing on Wang et al. (2021a)’ approach and the 6-item scale developed by Ng and Feldman (2010), we explored the extent to which coworkers perceive i-dealers (referred to as Peter below) in six dimensions (a level of pay, advancement opportunities, skill training, career development opportunities, a level of job security, support for personal problems). A sample item was “The organization promises Peter a level of job security that most employees in the department do not enjoy” (α = 0.918).

*Thriving at work (Time 2).* Drawing on the scale developed by Porath et al. (2012), the scale consists of five questions on each of the two dimensions of learning and vitality. A sample item of learning was “As time goes on, I learn more and more at work” (α = 0.831); A sample item of vitality was “I feel alive and vital at work” (α = 0.952).

*Innovation performance (Time 2).* Drawing on the scale developed by Janssen (2001), the scale consists of nine questions in three dimensions (idea generation, idea promotion, and idea realization). Sample items were “I always search out new working methods, techniques, or instruments; I always make important organizational members enthusiastic for innovative ideas; I try my best to introduce innovative ideas into the work environment in a systemic way” (α = 0.915).

*Employee humility (Time 1).* Drawing on the scale developed by Owens et al. (2013), the scale consists of nine questions in three dimensions (self-awareness, appreciation of employees’ strengths, and teachability). Sample items were “I acknowledge Peter has more knowledge and skills than me; I show appreciation for the unique contributions of Peter; I am willing to learn from Peter and employees” (α = 0.910).

*Control Variables (Time 1).* Consistent with the extant studies (Kong et al., 2020; Wang et al., 2021a), this study controlled demographic variables (gender, age, education, tenure, and monthly income).

## 4. Results

### 4.1. Common method biases test

This study adopted process control and statistical control to ensure the validity of the study results. The former was achieved by four means (i.e., questionnaire instructions, reverse question set, cross-formatting of items, and anonymous survey), while the latter was achieved by using the “Harman single-factor test” method of SPSS 26.0, which yielded an explained variance of the first factor was 26.469%; since it did not account for 50% of the total variance (Fuller et al., 2016), the common method bias was within acceptable limits.

### 4.2. Confirmatory factor analysis

To examine the discriminant validity of the variables, this study conducted a confirmatory factor analysis by using Mplus 7.4 software. Since the sample size was relatively small compared with the number of items, this study performs three-factor parceling for the five variables. Specifically, in order to reduce the parameter estimation bias (Bandalos, 2008), we (a) parceled the item with the largest and smallest factor loading as the first factor; (b) parceled the item with the second largest and second smallest loading as the second factor; and (c) parceled the remaining item as the third factor. The results of confirmatory factor analyses (see Table 1) showed that the five-factor model (χ^2^_[80]_ = 115.224, RMSEA = 0.042, CFI = 0.987, TLI = 0.983, SRMR = 0.034) fit the data better than any of alternative models, and this model met the ideal standard.

**Table 1:** Results of confirmatory factor analysis.

**Measurement Model**	***χ2***	***df***	***χ2/df***	**RMSEA**	**CFI**	**TLI**	**SRMR**
5-Factor model	115.224	80	1.440	0.042	0.987	0.983	0.034
4-Factor model	425.434	84	5.065	0.128	0.876	0.845	0.117
3-Factor model	928.920	87	10.677	0.198	0.695	0.631	0.179
2-Factor model	1688.566	89	18.973	0.269	0.420	0.315	0.215
1-Factor model	2237.508	90	24.861	0.310	0.221	0.091	0.245

5-Factor model (hypothesized model), 4-Factor model (CPEID and learning merged), 3-Factor model (CPEID and learning merged, vitality and innovation performance merged), 2-Factor model (CPEID, learning merged, vitality and innovation performance merged), 1-Factor model (CPEID, learning merged, vitality, innovation performance and humility merged). RMSEA, Root Mean Square Error of Approximation, CFI, Comparative Fit Index, TLI, Tucker-Lewis Index, SRMR, Standardized Root Mean Square Residual; and Source: Mplus 7.4 software analysis.

### 4.3. Descriptive statistics and correlations

Descriptive statistics and correlations of scales are displayed in Table 2. CPEID were positively related to coworker learning and coworker innovation performance (*r* = 0.206, 0.167, *p* < 0.01), and were negatively related to coworker vitality (*r* = −0.171, *p* < 0.01); Both coworker learning and coworker vitality were positively related to coworker innovation performance (*r* = 0.340, 0.185, *p* < 0.01); Coworker humility was positively related to both coworker learning and coworker vitality (*r* = 0.185, *p* < 0.01; 0.126, *p* < 0.05). Taken together, the hypothesized relationships between the variables were initially verified.

**Table 2:** Descriptive statistics and correlations.

Variable	*Mean*	*SD*	1	2	3	4	5	6	7	8	9
1.Gender	1.557	0.498									
2.Age	2.008	1.211	−0.249**								
3.Education	3.198	0.707	0.101	−0.248**							
4.Tenure	1.673	0.987	−0.230**	0.880**	−0.261**						
5.Monthly income	5.953	0.737	0.055	0.381**	0.001	0.368**					
6.CPEID	4.763	1.294	0.026	0.122	0.054	0.098	0.084				
7.Learning	4.984	1.202	−0.042	0.126*	−0.066	−0.008	0.084	0.206**			
8.Vitality	4.279	1.671	−0.001	0.067	−0.057	0.015	0.047	−0.171**	−0.148*		
9.Innovation performance	5.206	0.996	−0.131*	0.323**	−0.088	0.093	0.322**	0.167**	0.340**	0.185**	
10.Humility	4.821	1.117	−0.150*	0.182**	−0.059	0.150*	0.177**	0.006	0.185**	0.126*	0.259**

*n* = 248; *p < 0.05, **p < 0.01.

### 4.4. Test of hypotheses

By using Mplus 7.4 software for hypothesis testing, this study used Bootstrapping to replicate samples 5,000 times. The path coefficients of the mediator analysis are shown in Table 3. On the one hand, the results reported in Table 3 showed CPEID (a) positively predicted coworker learning (*β* = 0.192, *p* < 0.01); (b) negatively predicted coworker vitality (*β* = −0.222, *p* < 0.05); and (c) had a non-significant direct effect on coworker innovation performance (*β* = 0.044, *p* > 0.05). On the other hand, the results reported in Table 3 showed that both coworker learning and coworker vitality positively predicted coworker innovation performance. Therefore, both hypothesis 1 and hypothesis 2 were initially supported (i.e., both coworker learning and coworker vitality fully mediate the relationship between CPEID and coworker performance innovation).

**Table 3:** Path analysis of mediators.

**Path**	**Coefficient**	***SE***	**Boot 95% CI**
CPEID → Innovation performance	0.044	0.059	[−0.073, 0.165]
CPEID → Coworker learning	0.192**	0.069	[0.064, 0.334]
Coworker learning → innovation performance	0.526***	0.083	[0.360, 0.685]
CPEID → Coworker vitality	−0.222*	0.086	[−0.383, 0.041]
Coworker Vitality → Innovation performance	0.116**	0.039	[0.042, 0.195]

*n* = 248; SE: standard error; *p < 0.05, **p < 0.01, ***p < 0.001; and CI: confidence interval.

The results of the indirect effect analyses are shown in Table 4, which demonstrates the robustness of the study. The results showed that (a) the indirect effect value of CPEID affecting coworker innovation performance through coworker learning was 0.101 and the 95% confidence interval was [0.035, 0.189] (not including 0, significant); (b) the indirect effect value of CPEID affecting coworker innovation performance through coworker vitality was −0.026 and the 95% confidence interval was [−0.063, −0.005] (not including 0, significant); and (c) the total indirect effect value of CPEID affecting coworker innovation performance was 0.075 and the 95% confidence interval was [0.008, 0.164] (not including 0, significant). Therefore, hypothesis 1, hypothesis 2, and hypothesis 3 were initially supported.

**Table 4:** Results of mediating path analysis.

**Path**	**Stage**		**Effect**	
	**First**	**Second**	**Indirect**	**Total**
CPEID → Coworker learning → Innovation performance	0.192*** [0.064, 0.334]	0.526*** [0.360, 0.685]	0.101* [0.035, 0.189]	0.075* [0.008, 0.164]
CPEID → Coworker vitality → Innovation performance	−0.222* [−0.383, 0.041]	0.116** [0.042, 0.195]	−0.026* [−0.063, −0.005]	

*n* = 248; *p < 0.05; **p < 0.01; ***p < 0.001; CI confidence interval.

The path coefficients of the moderator analysis are shown in Table 5. The interaction term of CPEID and coworker humility positively predicted coworker learning (*β* = 0.208, *p* < 0.01), which supported Hypothesis 4a; the interaction term of CPEID and coworker humility had a non-significant effect on coworker vitality (*β* = −0.069, *p* = 0.411 > 0.05), which rejected hypothesis 4b (i.e., the moderating effect of Coworker humility between CPEID and coworker vitality was not significant).

**Table 5:** Path analysis of moderator.

**Path**	**Coefficient**	***SE***	**Boot 95% CI**
CPEID*Coworker humility → Coworker learning	0.208**	0.067	[0.072, 0.335]
CPEID*Coworker humility → Coworker vitality	−0.069	0.084	[−0.234, 0.091]

*n* = 248; **p < 0.01; SE: standard error; CI: confidence interval.

**Figure 2:**
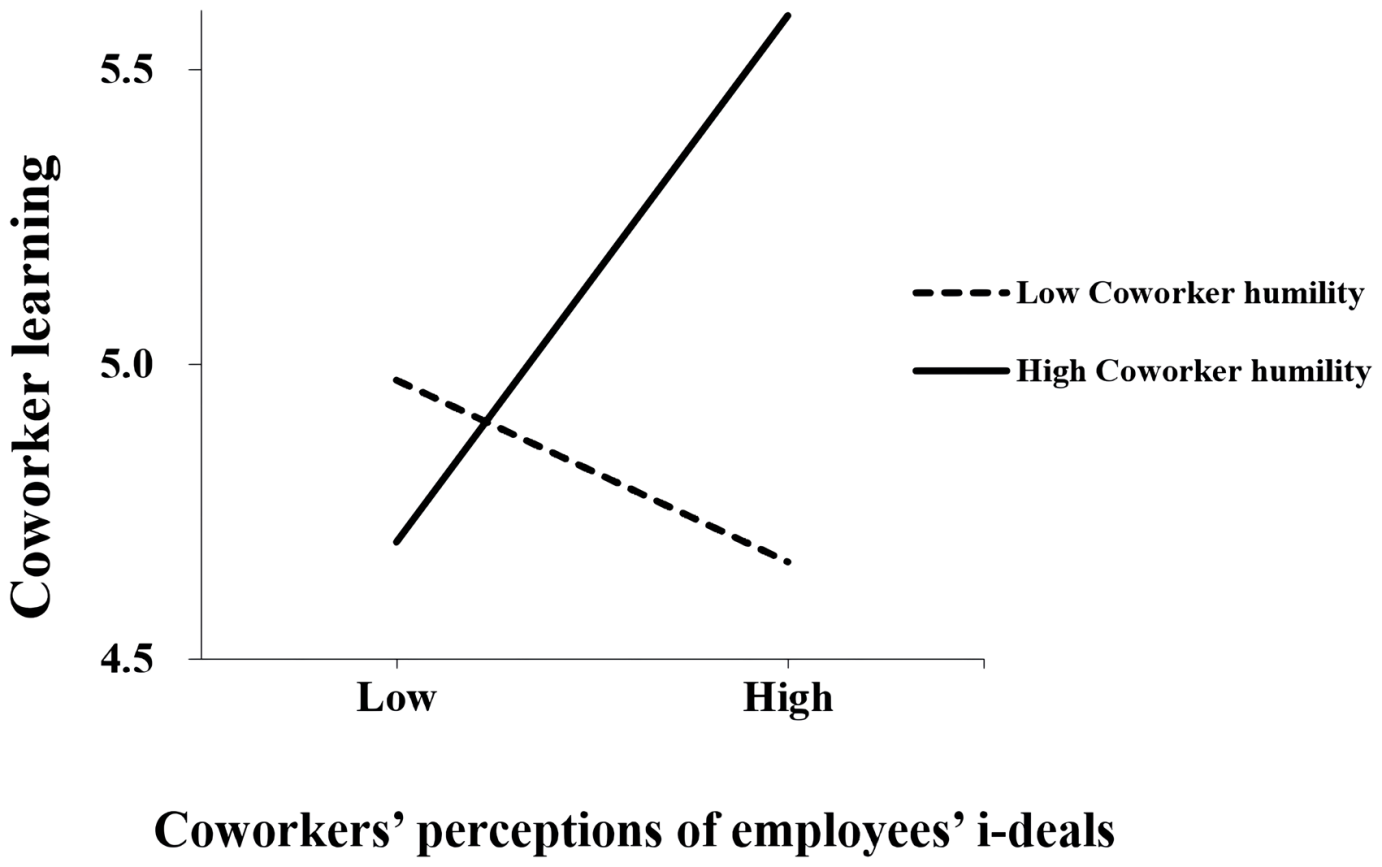
Moderating effect of coworker humility.

By adding and subtracting, respectively, one standard deviation from the mean of coworker humility, this study divided the sample into high and low groups to plot the moderating effect (see Figure 2). The results reported in Figure 2 showed that (a) at a low level of humility, the negative relationship between CPEID and coworker learning was not significant (γ = −0.119, *p* = 0.294 > 0.05); and (b) at a high-level humility, there was a significant positive relationship between CPEID and coworker learning (γ = 0.346, *p* < 0.001). Therefore, hypothesis 4a was further verified.

The results of the indirect effects of coworker learning at different levels of humility are shown in Table 6, which demonstrates the robustness of the study. When the level of humility is high, the indirect effect of CPEID on coworker innovation performance through coworker learning was 0.077, and the 95% confidence interval was [0.086, 0.291] (not including 0, significant); When the level of humility is low, the indirect effect of CPEID on coworker innovation performance through coworker learning was −0.027, and the 95% confidence interval was [−0.180, 0.053] (not including 0, significant); and the difference between the two was 0.104, and 95% confidence interval was [0.081, 0.422] (not including 0, significant). Taken together, the indirect effect of coworker learning was strengthened with increasing values of coworker humility, which supported hypothesis 5a.

**Table 6:** Results of indirect effect of moderated mediation.

**Moderator**	**Indirect effect**	***SE***	**95% CI**
Coworker humility	CPEID → Coworker learning → Innovation performance		
High (Mean + SD)	0.171**	0.052	[0.086, 0.291]
Low (Mean – SD)	−0.059	0.058	[−0.180, 0.053]
differences	0.230**	0.086	[0.081, 0.422]

*n* = 248; SE standard error; **p < 0.01; CI confidence interval.

## 5. Discussion

### 5.1. Conclusion

Based on the cognitive-affective processing system framework, this study examined the double-edged effect of CPEID on coworker innovation performance through (a) using coworker learning or coworker vitality as mediators, and (b) using coworker humility as a moderator. Based on a sample of 248 knowledge workers, this study had the following findings.

(1) In terms of cognition, granting i-deals showed that organizations are willing to invest in their employees, which inspires coworkers actively to learn and emulate i-dealers in order to improve coworker innovation performance. In terms of affection, owing to i-dealers occupying important organizational resources, coworkers’ organizational status is compromised, which will reduce coworker innovation performance by inhibiting coworker vitality. Integrating cognition and affection, the implementation of i-deals achieves a “win-win-win” management effect because CPEID have a positive total indirect effect on coworker innovation performance through coworker thriving at work.

(2) Coworker humility not only positively moderates the relationship between CPEID and coworker learning, but also reinforces the mediating role of coworker learning in the relationship between CPEID and coworker innovation performance. However, the moderating effect of coworker humility is not significant in the relationship between CPEID and coworker vitality. The reason may be that although coworkers with different levels of humility may differently interpret and evaluate the event of employees obtaining i-deals, CPEID undermine coworker vitality. Specifically, On the one hand, since coworkers with a high level of humility have a clear perception of the gap between themselves and i-dealers, they may believe that they have not obtained i-deals because of their lack of effort, which can lead to feelings of inferiority and thus unhappiness, anxiety, and depression (Smith et al., 1994); furthermore, when coworkers predict that they will not reach i-dealers’ ability level by doing their best, coworkers’ intrinsic motivation will be suppressed (Lockwood and Kunda, 1999), which further undermines their vitality. On the other hand, since coworkers with a low level of humility have a self-serving bias (Ma et al., 2022), they may believe that they do not receive i-deals because of managers’ bias (Wang et al., 2021b), which will lead to negative emotion (e.g., resentment, anger, self-worth denial). Accordingly, coworker humility does not significantly moderate the relationship between CPEID and vitality.

### 5.2. Theoretical implications

The theoretical implications of this study are the following:

Firstly, this study extends the theoretical perspective of i-deals from a bystander perspective. Most previous studies had focused on binary interaction scenarios (i.e., sender and receiver), neglecting the reactions of third parties (i.e., bystanders) to i-deals (Liao et al., 2016). Drawing upon the cognitive-affective processing system framework, we examine the mechanism of CPEID on coworker innovation performance. On the one hand, we integrate the positive and negative effects of i-deals implementation in order to provide a new theoretical perspective for i-deals research (Wang et al., 2021b); on the other hand, we provide empirical support for the hypothesis that the implementation of i-deals can achieve a “win-win-win” management effect (Lai et al., 2009).

Second, this study distinguishes the difference in the role of learning and vitality, and reveals the double-edged effect of CPEID in terms of cognitive and affective. We consider the fact that coworkers interpret and evaluate this event (employees get i-deals), which directly motivates their cognition and affection (Mischel and Shoda, 1995), and ultimately affects their distal outcomes (e.g., attitude, behavior, and performance). Therefore, we subdivide thriving at work into two dimensions: learning and vitality (Prem et al., 2017). The cognitive pathway extends the positive effects of i-deals. Specifically, unlike the mediating role of negative cognition (e.g., psychological contract violation, distributive injustice; Xiong et al., 2018; Marescaux et al., 2019), this study extends the positive effect of CPEID on coworker cognition to innovation performance by constructing a “CPEID-coworker learning-coworker innovation performance” action chain. The affective pathway is consistent with previous research (Ng, 2017; Kong et al., 2020; Wang et al., 2021a); namely, CPEID undermine coworker vitality by making them feel negative emotions, which in turn reduces their innovation performance.

In the end, this study examines the moderating role of employee humility, expanding the boundary conditions of i-deals. Considering that Chinese employees are deeply influenced by Chinese traditional culture of humility, this study focused on the different applicability of i-deals to differentiated individuals, exploring the moderating effect of employee humility on their cognitive units (Mischel and Shoda, 1995), i.e., whether the direct effect of CPEID on coworker learning and the indirect effect of CPEID on coworker innovation performance varies depending on their level of humility. This study (a) responds to Liao et al. (2016)’s call to focus on the role of individual characteristics of leaders and employees, (b) extends the boundary conditions for the positive role of CPEID in the Chinese context (Rousseau et al., 2009), and (c) provides new insights into the differential role of i-deals results based on cultural differences (Huo et al., 2014).

### 5.3. Practical implications

The practical implications of this study are as follows:

First, owing to the implementation of i-deals achieves a “win-win-win” management effect (e.g., coworkers will take initiatives—*via* viewing i-dealers as role models—to improve their innovation performance in order to obtain similar i-deals in the future), managers should take advantage of the positive effects of i-deals. Specifically, managers should (a) establish high-quality social and economic exchange relationships with employees (Lai et al., 2009), (b) convey the information to employees that organizations are willing to invest in talented employees, (c) encourage employees to improve their knowledge and skill levels, (d) increase the likelihood that employees obtain similar i-deals in the future (Ng and Lucianetti, 2016), (e) create a fair and equitable atmosphere as much as possible, and (f) motivate employees to correctly interpret organizational policies by increasing the openness and transparency of talent management policies.

Second, managers should pay attention to the psychological state of employees without i-deals and try to avoid the negative effects caused by the implementation of i-deals. As a differentiated HRM practice, CPEID will reduce coworker innovation performance by undermining coworker vitality. Therefore, managers should pay attention to the emotional reactions of coworkers. Specifically, when coworkers fall into negative emotions at work, managers should enhance the self-regulation ability and subfertility of coworkers through communication, positive feedback, and emotion regulation (Marescaux et al., 2021), which will mitigate the decrease in vitality and reduce the chances of subsequent negative behaviors.

In the end, managers should pay attention to differences in employees’ humility and take appropriate measures to impose positive and effective interventions on employees. This study showed that when coworker humility levels are high, CPEID increase coworker innovation performance by strengthening their learning. Since managers expressing humility can enhance employees’ humility to some extent (Zhong et al., 2019), managers can shape humble leadership through co-development behavior with employees. Managers should (a) actively communicate and interact with employees to help them establish correct self-perceptions, (b) create a good organizational learning atmosphere in order to dispel employees’ worries about exposing their own shortcomings, and (c) encourage employees to humbly and actively learn and seek advice.

### 5.4. Limitations and directions for future research

Although this study has some theoretical and practical significance, there are still aspects that need to be improved. Firstly, although this study used multiple time points to collect data in order to control endogenous, all data were obtained from the subjective reports of the employees who participated in the test, which resulted in the inability to verify causality. Therefore, future research could use experimental manipulation to further enhance the explanatory power of the model. Secondly, based on this study examining the double-edged effect of CPEID from a bystander perspective, future research could further explore other mechanisms of i-deals (e.g., developmental, task mechanisms). Thirdly, considering that this study examined the mediating role of coworker learning and coworker vitality based on the cognitive-affective processing system framework, future research could seek other cognitive and affective mechanisms based on other theories (e.g., transactional theory of stress and coping, and affective event theory). Finally, given that this study focused on the moderating effect of the personality trait (employee humility), future research could focus on the moderating effect of situational factors (e.g., transformational leadership, competitive climate) to dig deeper into the boundary conditions.

## Data availability statement

The original contributions presented in the study are included in the article/supplementary material, further inquiries can be directed to the corresponding author.

## Ethics statement

The studies involving human participants were reviewed and approved by Nanjing University, China. The patients/participants provided their written informed consent to participate in this study.

## Author contributions

CD: study conception and design, data collection, analysis of results, and manuscript preparation. LD: study design, manuscript preparation, and revision. JY: study conception and manuscript preparation. JC: manuscript finalization. All authors contributed to the article and approved the submitted version.

## Conflict of interest

The authors declare that the research was conducted in the absence of any commercial or financial relationships that could be construed as a potential conflict of interest.

## Publisher’s note

All claims expressed in this article are solely those of the authors and do not necessarily represent those of their affiliated organizations, or those of the publisher, the editors and the reviewers. Any product that may be evaluated in this article, or claim that may be made by its manufacturer, is not guaranteed or endorsed by the publisher.

## References

[ref1] Bakker A. B. Van Emmerik H. Van Riet P. (2008). How job demands, resources, and burnout predict objective performance: a constructive replication. Anxiety. Stress. Copin. 21, 309–324. doi: 10.1080/10615800801958637, PMID: 18612856

[ref2] Bandalos D. L. (2008). Is parceling really necessary? A comparison of results from item parceling and categorical variable methodology. Struct. Equ. Modeling 15, 211–240. doi: 10.1080/10705510801922340

[ref3] Brislin R. W. (1970). Back-translation for cross-cultural research. J. Cross-Cult. Psychol. 1, 185–216. doi: 10.1177/135910457000100301

[ref4] Campbell E. M. Liao H. Chuang A. Zhou J. Dong Y. (2017). Hot shots and cool reception? An expanded view of social consequences for high performers. J. Appl. Psychol. 102, 845–866. doi: 10.1037/apl0000183, PMID: 28191991

[ref5] Cohen-Charash Y. (2009). Episodic envy. J. Appl. Soc. Psychol. 39, 2128–2173. doi: 10.1111/j.1559-1816.2009.00519.x

[ref6] De Stobbeleir K. E. M. Ashford S. J. Buyens D. (2011). Self-regulation of creativity at work: the role of feedback-seeking behavior in creative performance. Acad. Manag. J. 54, 811–831. doi: 10.5465/amj.2011.64870144

[ref7] Exline J. J. Baumeister R. F. Bushman B. J. Campbell W. K. Finkel E. J. (2004). Too proud to let go: narcissistic entitlement as a barrier to forgiveness. J. Pers. Soc. Psychol. 87, 894–912. doi: 10.1037/0022-3514.87.6.894, PMID: 15598113

[ref8] Fuller C. M. Simmering M. J. Atinc G. Atinc Y. Babin B. J. (2016). Common methods variance detection in business research. J. Bus. Res. 69, 3192–3198. doi: 10.1016/j.jbusres.2015.12.008

[ref9] Grigoriou K. Rothaermel F. T. (2014). Structural micro-foundations of innovation: the role of relational stars. J. Manage. 40, 586–615. doi: 10.1177/0149206313513612

[ref10] Guerrero S. Challiol-Jeanblanc H. (2016). Idiosyncratic deals and helping behavior: the moderating role of i-deal opportunity for co-workers. J. Bus. Psychol. 31, 433–443. doi: 10.1007/s10869-015-9421-x

[ref11] Guo S. H. Hu Q. Q. (2022). Energetic learning: the effect of organizational identification and thriving at work on innovation performance. Manag. Rev. 34, 205–217. doi: 10.14120/j.cnki.cn11-5057/f.2022.01.011

[ref12] Ho V. T. Kong D. T. (2015). Exploring the signaling function of idiosyncratic deals and their interaction. Organ. Behav. Hum. Dec. 131, 149–161. doi: 10.1016/j.obhdp.2015.08.002

[ref13] Hornung S. Rousseau D. M. Glaser J. (2008). Creating flexible work arrangements through idiosyncratic deals. J. Appl. Psychol. 93, 655–664. doi: 10.1037/0021-9010.93.3.655, PMID: 18457493

[ref14] Huo W. Luo J. Tam K. L. (2014). Idiosyncratic deals and good citizens in China: the role of traditionality for recipients and their coworkers. Int. J. Hum. Resour. Manag. 25, 3157–3177. doi: 10.1080/09585192.2014.919949

[ref15] Janssen O. (2001). Fairness perceptions as a moderator in the curvilinear relationships between job demands, and job performance and job satisfaction. Acad. Manag. J. 44, 1039–1050. doi: 10.2307/3069447

[ref16] Jonas E. Mcgregor I. Klackl J. (2014). Threat and defense: From anxiety to approach. United States: Academic Press.

[ref17] Kong D. T. Ho V. T. Garg S. (2020). Employee and coworker idiosyncratic deals: implications for emotional exhaustion and deviant behaviors. J. Bus. Ethics 164, 593–609. doi: 10.1007/s10551-018-4033-9

[ref18] Lai L. Rousseau D. M. Chang K. T. T. (2009). Idiosyncratic deals: coworkers as interested third parties. J. Appl. Psychol. 94, 547–556. doi: 10.1037/a0013506, PMID: 19271808

[ref19] Lee K. Duffy M. K. (2019). A functional model of workplace envy and job performance: when do employees capitalize on envy by learning from envied targets? Acad. Manag. J. 62, 1085–1110. doi: 10.5465/amj.2016.1202

[ref20] Liao C. Wayne S. J. Rousseau D. M. (2016). Idiosyncratic deals in contemporary organizations: a qualitative and meta-analytical review. J. Organ. Behav. 37, S9–S29. doi: 10.1002/job.1959

[ref21] Lockwood P. Kunda Z. (1999). Increasing the salience of one’s best selves can undermine inspiration by outstanding role models. J. Pers. Soc. Psychol. 76, 214–228. doi: 10.1037/0022-3514.76.2.214, PMID: 10074706

[ref22] Ma J. Wang H. P. Yan Y. (2022). A jump is possible: when does envy of star employees make colleagues resentful and when does it inspire them to improve? J. Ind. Eng. Eng. Man. 36, 40–50. doi: 10.13587/j.cnki.jieem.2022.03.004

[ref23] Marescaux E. De Winne S. Rofcanin Y. (2021). Co-worker reactions to i-deals through the lens of social comparison: the role of fairness and emotions. Hum. Relat. 74, 329–353. doi: 10.1177/0018726719884103

[ref24] Marescaux E. De Winne S. Sels L. (2019). Idiosyncratic deals from a distributive justice perspective: examining co-workers’ voice behavior. J. Bus. Ethics 154, 263–281. doi: 10.1007/s10551-016-3400-7

[ref25] Mischel W. Shoda Y. (1995). A cognitive-affective system theory of personality: Reconceptualizing situations, dispositions, dynamics, and invariance in personality structure. Psychol. Rev. 102, 246–268. doi: 10.1037/0033-295X.102.2.246, PMID: 7740090

[ref26] Ng T. W. H. (2017). Can idiosyncratic deals promote perceptions of competitive climate, felt ostracism, and turnover? J. Vocat. Behav. 99, 118–131. doi: 10.1016/j.jvb.2017.01.004

[ref27] Ng T. W. H. Feldman D. C. (2010). Idiosyncratic deals and organizational commitment. J. Vocat. Behav. 76, 419–427. doi: 10.1016/j.jvb.2009.10.006

[ref28] Ng T. W. H. Lucianetti L. (2016). Goal striving, idiosyncratic deals, and job behavior: goal striving and idiosyncratic deals. J. Organ. Behav. 37, 41–60. doi: 10.1002/job.2023

[ref29] Owens B. P. Hekman D. R. (2012). Modeling how to grow: an inductive examination of humble leader behaviors, contingencies, and outcomes. Acad. Manag. J. 55, 787–818. doi: 10.5465/amj.2010.0441

[ref30] Owens B. P. Johnson M. D. Mitchell T. R. (2013). Expressed humility in organizations: implications for performance, teams, and leadership. Organ. Sci. 24, 1517–1538. doi: 10.1287/orsc.1120.0795

[ref31] Pan J. Zheng X. (. J.). Xu H. (. H.). Li J. (. K.). Lam C. K. (2021). What if my coworker builds a better LMX? The roles of envy and coworker pride for the relationships of LMX social comparison with learning and undermining. J. Organ. Behav. 42, 1144–1167. doi: 10.1002/job.2549

[ref32] Pierce J. L. Gardner D. G. (2004). Self-esteem within the work and organizational context: a review of the organization-based self-esteem literature. J. Manage. 30, 591–622. doi: 10.1016/j.jm.2003.10.001

[ref33] Porath C. Spreitzer G. Gibson C. Garnett F. G. (2012). Thriving at work: toward its measurement, construct validation, and theoretical refinement. J. Organiz. Behav. 33, 250–275. doi: 10.1002/job.756

[ref34] Prem R. Ohly S. Kubicek B. Korunka C. (2017). Thriving on challenge stressors? Exploring time pressure and learning demands as antecedents of thriving at work. J. Organiz. Behav. 38, 108–123. doi: 10.1002/job.2115, PMID: 28133415 PMC5244684

[ref35] Rousseau D. M. (2001). Flexibility versus fairness? Organ. Dyn. 29, 260–273. doi: 10.1016/S0090-2616(01)00032-8

[ref36] Rousseau D. M. Ho V. T. Greenberg J. (2006). I-deals: idiosyncratic terms in employment relationships. Acad. Manag. Rev. 31, 977–994. doi: 10.5465/amr.2006.22527470

[ref37] Rousseau D. M. Hornung S. Kim T. G. (2009). Idiosyncratic deals: testing propositions on timing, content, and the employment relationship. J. Vocat. Behav. 74, 338–348. doi: 10.1016/j.jvb.2009.02.004

[ref38] Rousseau D. M. Tomprou M. Simosi M. (2016). Negotiating flexible and fair idiosyncratic deals (i-deals). Organ. Dyn. 45, 185–196. doi: 10.1016/j.orgdyn.2016.07.004

[ref39] Schmitt M. Baumert A. Gollwitzer M. Maes J. (2010). The justice sensitivity inventory: factorial validity, location in the personality facet space, demographic pattern, and normative data. Soc. Just. Res. 23, 211–238. doi: 10.1007/s11211-010-0115-2

[ref40] Smith R. H. Parrott W. G. Ozer D. Moniz A. (1994). Subjective injustice and inferiority as predictors of hostile and depressive feelings in envy. Pers. Soc. Psychol. B. 20, 705–711. doi: 10.1177/0146167294206008

[ref41] Spreitzer G. Sutcliffe K. Dutton J. Sonenshein S. Grant A. M. (2005). A socially embedded model of thriving at work. Organ. Sci. 16, 537–549. doi: 10.1287/orsc.1050.0153

[ref42] Vidyarthi P. R. Singh S. Erdogan B. Chaudhry A. Posthuma R. Anand S. (2016). Individual deals within teams: investigating the role of relative i-deals for employee performance. J. Appl. Psychol. 101, 1536–1552. doi: 10.1037/apl0000145, PMID: 27513681

[ref43] Wang L. L. Long L. R. Zhang Y. (2021b). The relationship between newcomers’ l-deals and coworkers’ ostracism and self-improvement: the mediating role of envy and the moderating role of organizational overall justice. Manag. Rev. 33, 234–244. doi: 10.14120/j.cnki.cn11-5057/f.2021.08.020

[ref44] Wang L. L. Zhang F. Y. Tu Y. Zhang X. (2021a). The double-edged sword effect of idiosyncratic deals on bystanders. Hum. Resour. Dev. China. 38, 63–75. doi: 10.16471/j.cnki.11-2822/c.2021.9.005

[ref45] Xiong J. Ye M. L. Chen Y. S. (2018). Exploring the effect of coworkers’ idiosyncratic deals on employees work withdrawal behavior: based on the perspective of equity theory. J. Psychol. Sci. 41, 929–935. doi: 10.16719/j.cnki.1671-6981.20180425

[ref46] Zhong J. Zhang L. Li P. Zhang D. Z. (2019). Can leader humility enhance employee wellbeing? The mediating role of employee humility. Leadership. Org. Dev. J. 41, 19–36. doi: 10.1108/LODJ-03-2019-0124

